# Production of Avaroferrin and Putrebactin by Heterologous Expression of a Deep-Sea Metagenomic DNA

**DOI:** 10.3390/md12094799

**Published:** 2014-09-12

**Authors:** Masaki J. Fujita, Ryuichi Sakai

**Affiliations:** 1 Creative Research Institution, Hokkaido University, 3-1-1 Minato-cho, Hakodate, Hokkaido 041-8611, Japan; 2 Faculty of Fisheries Sciences, Hokkaido University, 3-1-1 Minato-cho, Hakodate, Hokkaido 041-8611, Japan; E-Mail: ryu.sakai@fish.hokudai.ac.jp

**Keywords:** metagenome, siderophore, avaroferrin, biosynthesis, heterologous expression

## Abstract

The siderophore avaroferrin (**1**), an inhibitor of *Vibrio* swarming that was recently identified in *Shewanella algae* B516, was produced by heterologous expression of the biosynthetic gene cluster cloned from a deep-sea sediment metagenomic DNA, together with two analogues, bisucaberin (**2**) and putrebactin (**3**). Avaroferrin (**1**) is a macrocyclic heterodimer of *N*-hydroxy-*N*-succinyl cadaverine (**4**) and *N*-hydroxy-*N*-succinyl-putrescine (**5**), whereas analogues **2** and **3** are homodimers of **4** and **5**, respectively. Heterologous expression of two other related genes from culturable marine bacteria resulted in production of compounds **1**–**3**, but in quite different proportions compared with production through expression of the metagenomic DNA.

## 1. Introduction

Metagenomics enables genomic DNA to be obtained directly from environmental samples without any microbial isolation and cultivation steps [1]. This method facilitates the isolation of biosynthetic genes from previously unreachable genetic pools, such as uncultivated environmental microorganisms or symbionts of marine invertebrates, which are difficult to culture in most cases [2].

**Figure 1:**
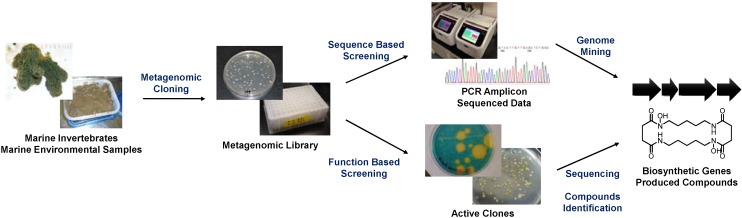
General scheme of metagenomic studies in the field of marine natural product chemistry.

Currently, two major metagenomic approaches are used in the field of marine natural product chemistry: sequence-based and function-based methods (Figure 1). The sequence-based approach targets conserved biosynthetic genes and searches for homologous sequences within a large set of sequence data or mines for genes from a random DNA pool using PCR amplification. This method is highly advantageous for searches involving well-studied gene classes, such as those encoding biosynthetic machineries of polyketides, non-ribosomal peptides, and post-translationally modified ribosomal peptides. Remarkable advances in targeting such genes have been made recently following the development of next-generation sequencing techniques [3,4,5,6]. The sequence-based approach has thus become the standard method for mining the enormous biosynthetic gene clusters of marine symbiotic bacteria, which are thought to be responsible for the production of various secondary metabolites of marine invertebrates. 

The function-based metagenomic approach involves the isolation of biosynthetic genes from a random metagenomic library using specific selection methods (e.g., biological assays) [2]. The function-based approach relies on the ability of a heterologous host microorganism (*E. coli* in most cases) to produce functional molecules that are detectable by the selection method employed. Therefore, only genes that can produce the final product are selected. To date, several marine metagenomic products have been heterologously produced using this approach, such as halichrome A and indole-porphyrin hybrids from the marine sponges *Halichondria okadai* [7] and *Discodermia calyx* [8], respectively. 

**Figure 2:**
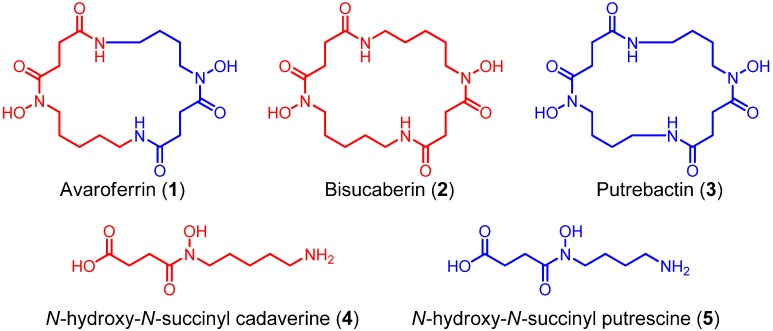
Structures of *N*-hydroxy-*N*-succinyl diamine (HSD)-based siderophores.

Using a functional metagenomic approach and marine samples, we previously produced several siderophores, including the polycarboxylate-type siderophore vibrioferrin [9] and the hydroxamate-type siderophore bisucaberin (**2**) [10,11], the latter of which is a macrocyclic dimer of *N*-hydroxy-*N*-succinyl cadaverine (**4**, HSC, Figure 2). Recently, we found that the substrate selectivity of the bisucaberin (**2**) biosynthesis enzymes MbsA, B, and C encoded in the cloned metagenomic DNA is broader than would be expected, because both the bisucaberin (**2**) precursor HSC (**4**) and another potential precursor, *N*-hydroxy-*N*-succinyl putrescine (HSP, **5**), were detected in the culture broth of the *mbsA-C* clone [12], suggesting the presence of additional products. We were thus interested in the expandable nature of the marine metagenome, which enables the production of a diverse array of molecules from a single set of genes. Here, we report the results of our detailed analysis of the metabolites from a metagenomic clone and a comparison of the catalytic features of the enzymes with related biosynthetic enzymes from other marine bacteria. Our results demonstrate the potential diversity of the marine metagenome and the usefulness of the function-based approach to analyze genes with unknown functions.

## 2. Results and Discussion

**Figure 3:**
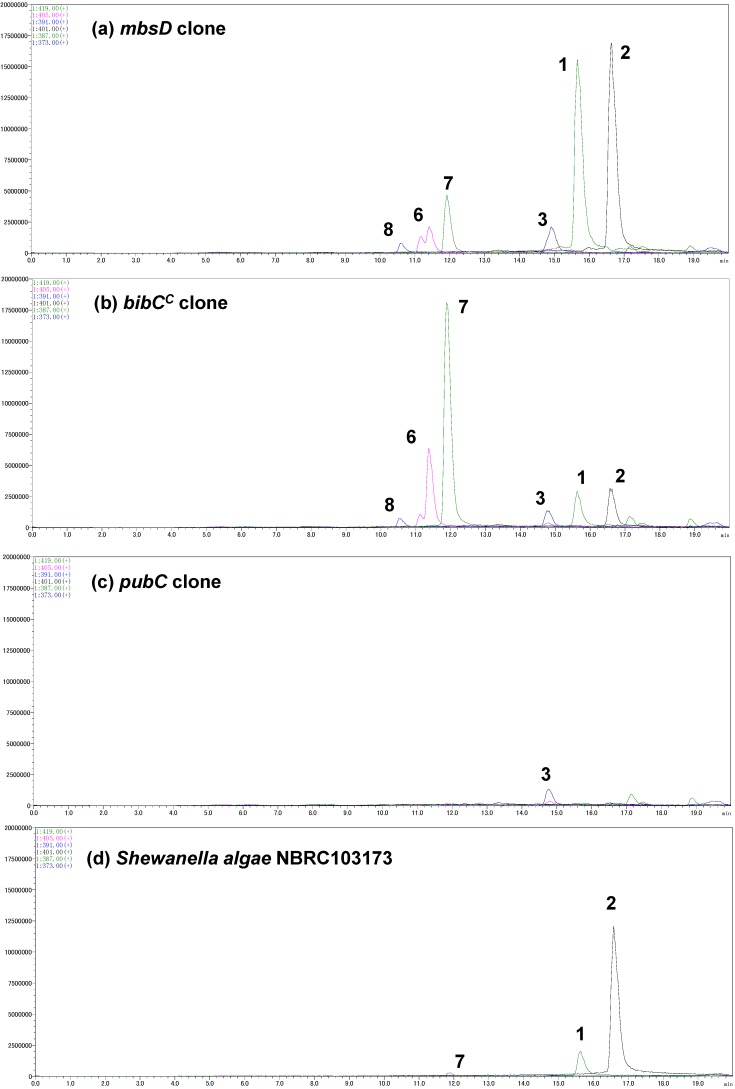
Selected ion current LC-MS chromatograms of the culture broths of the marine metagenome-derived *mbsA-D* clone (**a**); fusion gene cluster clones (**b**,**c**); and *Shewanella algae* NBRC103173 (**d**).

A gene cluster (*mbsA-D*) encoding enzymes for the biosynthesis of the siderophore bisucaberin (**2**) was cloned from a metagenomic library derived from a deep-sea sediment sample collected off the Tokara Islands [11]. In a previous study, we reported the isolation of **2**, an HSC (**4**) dimer, as the sole product from the cultured clone. We later realized, however, that MbsA, B, and C are capable of supplying not only HSC (**4**) but also HSP (**5**) [12]. This result suggested to us that other macrocyclic products containing HSP (**5**) as a precursor can be biosynthesized from the same gene set. To examine this possibility, the biosynthetic gene cluster *mbsA-D* was again expressed in *E. coli* and the culture broth was analyzed using LC-MS (Figure 3a). We not only found the expected major product, **2**, but also putrebactin (**3**; *m/z* 373) [13], avaroferrin (**1**; *m/z* 387; a heterodimer of HSC (**4**) and HSP (**5**)), bisucaberin B (**7**; *m/z* 419) [14], and three other putative linear intermediates (**6a**, ** 6b ** [*m/z* 405], and **8** [*m/z* 391]). Isolation of macrocyclic compounds **2** and **3** from several marine bacteria was reported decades ago [10,13], whereas compound **1** was isolated from a cultured marine bacterium, *Shewanella algae* B516, only recently during preparation of this manuscript [15]. Compounds **1** and **3** were purified by HPLC and their structures confirmed unambiguously by spectral analysis. This is the first report of the heterologous production of **1** and **3**, and the deep-sea metagenomic clone encoding *MbsA-D* represents the first cluster of avaroferrin (**1**) biosynthetic enzymes thus far experimentally confirmed. 

Avaroferrin (**1**) was reported to inhibit the swarming of *Vibrio alginolyticus* B522 [15]. In our previous study, we reported the isolation of **2** as the sole product of the same metagenomic cluster. Our present results, however, clearly show that this gene set can produce more than one siderophore. Differences in the results between those studies are largely due to difference in the isolation and detection method, that is; we purified **2** simply by recrystallization in the previous study, but this time we thoroughly investigated the gene product by utilizing LC-MS, to characterize the versatile nature of the enzymes. The ferric ion-chelating activities of compounds **1**–**3** as determined by a chrome azurol S titration assay [16] were similar (EC_50_ values: 4.0, 3.5, and 4.8 μM, respectively, see Supplementary Information), suggesting that all three compounds function as ferric ion acquisition agents for the original producer. 

The present results provide some insights into the functions of the enzymes involved in the biosynthesis of *N*-hydroxy-*N*-succinyl diamine (HSD)-based siderophores. In the previously proposed HSD-based siderophore biosynthesis pathway, the first three enzymes A–C catalyze successive reactions leading from lysine and ornithine to the HSDs [17]. Subsequently, the fourth enzyme D catalyzes both the oligomerization of the HSDs and final macrocyclization. The fact that the MbsA-D enzyme set cloned into *E. coli* produced the cyclic products **1**–**3** and the linear intermediates **6**–**8** simultaneously strongly suggests that MbsD can utilize both **4** and **5** as immediate precursors and condense them in both a homo- and hetero-meric manner. To test this hypothesis, we constructed two fused gene sets, comprising *mbsA-C* plus either *bibC^C^* or *pubC*, both of which encode condensation enzymes corresponding to *mbsD* from the biosynthetic systems of **2** [18] and **3** [19] from the siderophore-producing marine bacteria *Aliivibrio salmonicida* LFI1238 and *Shewanella* sp. MR-4, respectively. These chimeric gene clusters allowed for functional testing of the fourth enzymes, as the metagenomic MbsA-C enzymes would supply the common monomers **4** and **5**, whereas the fourth enzymes would catalyze the oligomerization and macrocyclization of the monomers when expressed in the heterologous host. 

**Figure 4:**
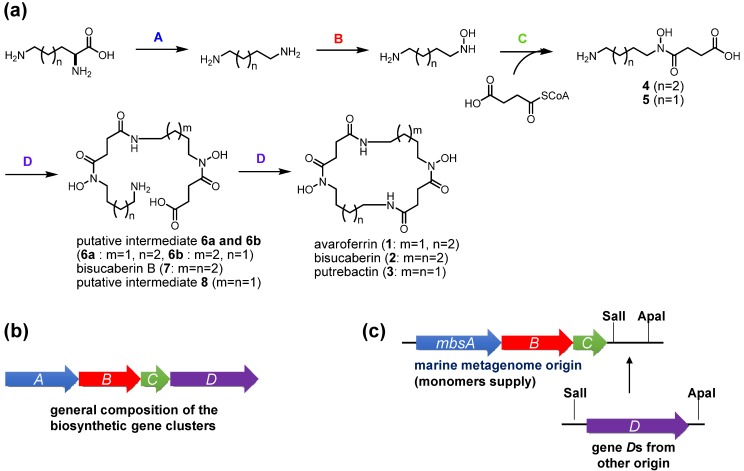
Proposed general scheme for the biosynthesis of HSD-based siderophores (**a**) and composition of their biosynthetic gene clusters (**b**); The fusion gene cluster system involving metagenome-derived *mbsA-C* and *genes D* of other origin is also shown (**c**).

The codon-optimized *bibC^C^* and *pubC* genes were chemically synthesized and inserted downstream of *mbsC* using the cassette method (Figure 4c). Transformants of these fusion gene clusters were cultured and the supernatants were subjected to LC-MS analysis (Figure 3b,c). The clone containing *bibC^C^* produced compounds **1** and **2**, together with a small amount of **3**, in a 2:2:1 ratio. However, the major metabolites of this clone were acyclic intermediates (compounds **6**–**8**). Of note, the accumulation of bisucaberin B (**7**) was six-times that of the macrocyclic counterpart **2**. These results suggested that BibC^C^ is also capable of producing avaroferrin (**1**) and putrebactin (**3**), but its macrocyclization activity is not as potent as that of MbsD, at least in the heterologous host *E. coli*. In the case of the *pubC* clone, the most abundant siderophore produced was putrebactin (**3**), and aside from unknown background peaks, no detectable amounts of other metabolites were observed in the LC-MS chromatogram. Overall siderophore production in this case was around 3% compared with that of the *mbsD* and *bibC^C^* clones, suggesting that the inherent functionality of PubC was not expressed in the heterologous host.

We also analyzed the siderophores from *Shewanella algae* NBRC103173, which is another strain of avaroferrin (**1**)-producing bacterium (Figure 3d). Interestingly, this *S. algae* strain produced mainly bisucaberin (**2**) and a small amount of avaroferrin (**1**) but did not produce detectable amounts of **3**. This production pattern was quite different from that reported for *S. algae* strain B516, which produced compounds **1**–**3** at a 1:2:1 ratio [15]. Although the culture conditions were not completely the same, one can suggest, judging from the large difference of the production profiles, that these enzyme sets can be one of the keys to add metabolic diversity even within the same species. 

The amino acid sequence identity for the three condensation enzymes examined, MbsD, BibC^C^, and PubC, is around 60%, but it is unclear which amino acid residues are responsible for the differences in enzyme function. In addition, the molecular mechanisms of the enzymatic oligomerization and macrocyclization reactions are still largely unknown. Structural-biological analyses of these enzymes as well as other related biosynthetic pathways are in progress. 

The results of the present study demonstrating the production of multiple siderophores by one enzyme system reveal the remarkable versatility of the biosynthetic enzymes of HSD-based siderophores. The unique property of these enzymes might be advantageous to producers in competitive microbial communities. For example, some *Vibrio* species are known to utilize exogenous siderophores produced by other bacteria in the community by expressing siderophore-specific receptors [20]. This phenomenon, termed *siderophore piracy*, may explain discrete differences in the ecological functions of the structurally related siderophores **1**–**3**, and is illustrated by *V. alginolyticus* B522, which is capable of “stealing” **2** and **3** (but not **1**) using specific receptors (siderophore uptake system) [15]. Thus, the ability to produce multiple siderophores could represent a strategy to avoid siderophore piracy. Our study demonstrates that a slight change in the sequence of a siderophore biosynthesis gene can significantly modify the enzyme’s properties and thus have a large impact on siderophore production.

The *mbsA-D* metagenomic siderophore biosynthesis gene cluster may have a common ancestor with that of * Shewanella* sp. or related strains, based on sequence similarity with the putative avaroferrin (**1**) biosynthesis gene cluster from *S. algae* B516 [15]. However, the apparent differences in catalytic function of the *mbsA-D* cluster compared with other related enzymes thus far isolated from culturable bacteria demonstrate the power of the function-based metagenomic approach, which may result in the discovery of completely unexpected functions of environmental genomes. 

## 3. Experimental Section 

### 3.1. General Experimental Procedures

Low- and high-resolution ESI mass spectra were measured on an Exactive mass spectrometer (Thermo Scientific, Waltham, MA, USA). NMR spectra were recorded on an ECP-400 NMR spectrometer (JEOL, Tokyo, Japan) at 400 MHz for ^1^H and 100 MHz for ^13^C in CD_3_OD and DMSO-*d*_6_ (1:1) as a solvent. Chemical shifts of ^1^H and ^13^C in NMR spectra were referenced to the solvent peaks: δ_H_ 3.30 and δ_C_ 49.0 for CD_3_OD. UV absorption in the CAS assay was measured on a SpectraMax M2 microplate reader (Molecular Devices, Sunnyvale, CA, USA). Preparative HPLC was done with a Prominence HPLC system equipped with a photodiode array detector (Shimadzu, Kyoto, Japan). LC-MS analyses were done with an LCMS-8040 LC-MS system equipped with a Prominence HPLC system (Shimadzu). DNA sequences were determined with a BigDye terminator cycle sequencing kit (Thermo Fisher Scientific) and a 3130*xl* Genetic Analyzer (Thermo Fisher Scientific). For DNA cloning, NEB 10-beta-competent *E. coli* cells (New England BioLabs, Ipswich, MA, USA) were used. Electroporation was done with a MicroPulser electroporator (Bio-Rad, Hercules, CA, USA). Oligo DNAs for cloning and DNA sequencing were purchased from Hokkaido System Science (Sapporo, Japan). A GeneAtlas thermal cycler (Astec, Fukuoka, Japan) and a KOD Plus Neo PCR kit (Toyobo, Osaka, Japan) were used to amplify DNA fragments. All chemicals were purchased from Wako Pure Chemical Industries (Osaka, Japan), Nacalai Tesque (Kyoto, Japan), or Takara Bio (Shiga, Japan) except for those specifically mentioned.

### 3.2. Construction and Screening of a Metagenomic Library 

Detailed methods for construction of a deep sea sediment metagenomic library and cloning of *mbs* gene cluster have been reported in the previous paper [11]. Briefly, deep sea sediment samples were collected by dredging (150–1000 m) and directly poured into flasks containing sterilized sea water, and then vigorously shaken to wash out bacteria cells. The suspension was fractionated by stepwise centrifugation to afford cell pellet. It was washed with TES buffer (20 mM Tris-HCl (pH 8.0), 100 mM EDTA (pH 8.0), 50 mM NaCl, 25% sucrose) and centrifuged, then re-suspended in TES buffer. Cells were lysed with SDS and proteinase K, and then treated with 5 M NaCl aqueous solution and 10% cetyltrimethylammonium bromide (CTAB). The resulting DNA solution was cleaned up by phenol-chloroform treatment, followed by precipitation by addition of isopropanol to afford crude metagenomic DNA. It was size fractionated by agarose gel electrophoresis. The recovered large molecular weight DNA was blunt-ended and ligated into fosmid vector. This was transformed into *E. coli* and plated on the LB agar containing chloramphenicol to construct a metagenomic library. The *mbs* cluster containing clone (*mbsA-D*, DNA Data Bank of Japan; AB643578) was obtained based on the siderophore production activity using chrome azurol S assay [16].

### 3.3. Artificial Genes

Two artificial genes whose sequence was optimized for *E. coli* expression were chemically synthesized by FASMAC (Kanagawa, Japan). Recognition sites for restriction enzymes SalI and ApaI were added at the 5′ and the 3′ ends, respectively (for sequence, see Supplemental Information). 

*pubC*: 1887 bp DNA fragment encoding 629 amino acids of the putrebactin biosynthetic enzyme PubC (*Shewanella* sp. MR-4; NCBI referenced sequence: YP_733587). 

*bibC^C^*: 1857 bp DNA fragment encoding 619 amino acids of the C-terminal portion of the biosynthetic enzyme BibC (*Aliivibrio salmonicida* LFI1238; NCBI referenced sequence: YP_002261686). 

### 3.4. Preparation of Siderophore Producing Fusion Gene Cluster Clones

A DNA fragment encoding metagenomic bisucaberin biosynthetic enzymes, MbsA-C, was amplified from the bisucaberin biosynthetic gene cluster from a deep sea metagenome (accession number: AB643578) by PCR using a following primer set: forward, 5′-ACGTCTAGATDGATCGCTCTCAACTCAGCC-3′ and reverse, 5′-TTTGTCGACTCATTGTGTGGCTCCTGTTGC-3′ (underlined sequences show XbaI and SalI recognition sites, respectively). Polymerase chain reaction was done following the protocol provided by the manufacturer, initial denaturation at 95 °C for 2 min, followed by 30 cycles 30 s at 92 °C, 30 s at 60 °C, and 2.5 min at 72 °C. The amplicon was cut by XbaI and SalI, purified, and ligated into pBCSK+ phagemid vector (Agilent Technologies, Santa Clara, CA, USA) to form a monomers supply clone. Purchased artificial genes, *pubC* and *bibC^C^*, were digested by SalI and ApaI, then ligated into monomer supply clone downstream of *mbsA-C* with T4 DNA ligase (Takara Bio) to produce the fusion gene clusters. They were transformed into NEB 10-beta-competent *E. coli* cells by electroporation, and then spread onto LB agar plates supplemented with chloramphenicol to obtain the fusion cluster clones (*mbsA-C* + *pubC*, *mbsA-C* + *bibC^C^*). 

### 3.5. Production and Identification of Avaroferrin and Putrebactin 

The metagenome derived *mbsA-D* cluster clone was pre-cultured overnight in LB medium containing chloramphenicol and then inoculated into four 1-L flasks containing 400 mL of LB medium supplemented with 30 μg/mL chloramphenicol and 0.1 mM isopropyl β-thiogalactopyranoside (IPTG). They were incubated at 37 °C for 3 day with shaking at 225 rpm. After centrifugation, the siderophore active molecules in the supernatant were adsorbed onto C18 resin, then eluted with stepwise aqueous-MeOH gradient system (0%–100% MeOH). Fractions eluted with 20%–50% MeOH were combined and fractionated with Sephadex G-10 gel filtration with water. The fraction containing the mixture of siderophores was further separated by Sephadex LH-20 column chromatography with 50% aqueous MeOH to afford siderophore concentrated fraction. A part of the fraction (600 mL culture equivalent) was purified by repetitive C18 reversed phase HPLC (Inertsil ODS-3, GL Sciences, Tokyo) with an aqueous MeOH linear gradient (first HPLC: 20%–60% MeOH, second HPLC: 30%–44% over 30 min) to yield pure avaroferrin (**2**, 6.9 mg from 600 mL). 

Putrebactin (**3**) was also isolated in a similar manner to that described above. Total 8.0 L of cultured medium was solid phase extracted with C18 resin, then subjected to the two steps of C18 column chromatography with aqueous MeOH. Compound **3** containing fractions (eluted with 10%–20% MeOH) were combined and subsequently fractionated by a Sephadex G-10 column with water containing 0.2% acetic acid, then C18 HPLC (20%–60% MeOH) to yield putrebactin (**3**, 9.5 mg from 8.0 L)

Avaroferrin (**1**): white amorphous; HR-ESIMS, 409.20543 (calcd. for C_17_H_30_O_6_N_4_Na, −0.8 ppm); NMR data (CD_3_OD: DMSO-*d*_6_ = 1:1) δ_H _3.65 (t, *J* = 6.4 Hz, 2H), 3.64 (t, *J* = 6.4 Hz, 2H), 3.19 (t, *J* = 6.4 Hz, 4H), 2.77 (t, *J* = 6.4 Hz, 4H), 2.47 (t, *J* = 6.4 Hz, 4H), 1.67(quint., *J* = 7.2 Hz, 4H), 1.52 (m, 4H), 1.34 (m, 2H); δ_C _173.8 (4C), 47.4 (1C)^a^, 47.0 (1C), 38.6 (2C)^a^, 30.6 (2C), 28.3 (1C), 28.0 (2C), 26.3 (1C), 25.6 (1C), 24.0 (1C), 22.8 (1C) (a: carbon chemical shifts were determined from the HSQC spectrum due to peak overlapping on solvent signals).

Putrebactin (**3**): colorless residue; HR-ESIMS, 395.19011 (calcd. for C_16_H_28_O_6_N_4_Na, −0.01 ppm); NMR data (CD_3_OD: DMSO-*d*_6_ = 1:1) δ_H _3.63 (t, *J* = 6.8 Hz, 4H), 3.19 (t, *J* = 6.8 Hz, 4H), 2.77 (t, *J* = 6.4 Hz, 4H), 2.46 (t, *J* = 6.4 Hz, 4H), 1.65 (broad t., *J* = 6.8 Hz, 4H), 1.52 (m, 4H), (carbon NMR signals were not detected due to too low solubility in any solvents tested).

### 3.6. LC-MS Analysis

Siderophore producing clones were cultured at 30 °C for 3 day with shaking at 225 rpm in LB medium, containing 30 μg/mL of chloramphenicol and 0.1 mM IPTG. The resulting culture medium was mixed with an equal volume of MeOH, then centrifuged to remove insoluble materials. A portion of the supernatant was analyzed by LC-MS (column, Inertsil ODS-3, 2 × 100 mm; solvents, 0%–60% aqueous-MeOH linear gradient system with 0.2% AcOH; flow rate, 0.2 mL/min; detection, SIM at *m/z* 419, 405, 401, 391, 387, and 373. Marine bacterium *Shewanella algae* NBRC103173 was cultured in the sea water based medium (0.5 g trypton, 0.05 g yeast extract in 1.0 L sea water)) at 30 °C for 3 day with shaking at 225 rpm. Culture broth was subjected to the LC-MS analysis with the same method. Peak area of the mass chromatogram was quantitated using LC-MS solution software (Shimadzu, Kyoto, Japan). 

### 3.7. Chrome Azurol S Assay

Test sample solutions (100 μL) were mixed with equal volume of CAS assay solution (0.6 mM cetyltrimethylammonium bromide, 15 μM FeCl_3_, 150 mM CAS, 0.5 M anhydrous piperazine, 0.75 M HCl) in a 96 well microplate, and kept at room temperature for 4 h, and then the absorption at 630 nm was measured by microplate reader. Experiments were performed in triplicate. 

## 4. Conclusions 

Two additional siderophores, avaroferrin (**1**) and putrebactin (**3**), were heterologously produced together with previously reported bisucaberin (**2**) using a marine metagenome-derived siderophore biosynthesis gene cluster. Compound **1** was only recently identified in a marine bacterium, *Shewanella algae* B516, and was shown to inhibit the swarming behavior of *Vibrio parahaemolyticus* B522. LC-MS analyses of the fusion gene cluster clones revealed that other homologous enzymes can catalyze the production of compound **1**, but the production ratio of compounds **1**–**3** was quite different. These results suggest that a metagenomic approach is a practical way to isolate enzymes with characteristic catalytic properties and that it might be possible to isolate previously unidentified biosynthetic enzymes from uncultivated bacteria. 

## Supplementary Files

Supplementary File 1

## References

[1] Rondon M.R., August P.R., Bettermann A.D., Brady S.F., Grossman T.H., Liles M.R., Loiacono K.A., Lynch B.A., MacNeil I.A., Minor C. (2000). Cloning the soil metagenome: A strategy for accessing the genetic and functional diversity of uncultured microorganisms. Appl. Environ. Microbiol..

[2] Kennedy J., Flemer B., Jackson S.A., Lejon D.P.H., Morrissey J.P., O’Gara F., Dobson A.D.W. (2010). Marine metagenomics: New tools for the study and exploitation of marine microbial metabolism. Mar. Drugs.

[3] Piel J., Hui D., Wen G., Butzke D., Platzer M., Fusetani N., Matsunaga S. (2004). Antitumor polyketide biosynthesis by an uncultivated bacterial symbiont of the marine sponge theonella swinhoei. Proc. Natl. Acad. Sci. USA.

[4] Freeman M.F., Gurgui C., Helf M.J., Morinaka B.I., Uria A.R., Oldham N.J., Sahl H.-G., Matsunaga S., Piel J. (2012). Metagenome mining reveals polytheonamides as posttranslationally modified ribosomal peptides. Science (Wash.).

[5] Wilson M.C., Mori T., Rueckert C., Uria A.R., Helf M.J., Takada K., Gernert C., Steffens U.A.E., Heycke N., Schmitt S. (2014). An environmental bacterial taxon with a large and distinct metabolic repertoire. Nature (Lond.).

[6] Sudek S., Lopanik N.B., Waggoner L.E., Hildebrand M., Anderson C., Liu H., Patel A., Sherman D.H., Haygood M.G. (2007). Identification of the putative bryostatin polyketide synthase gene cluster from “Candidatus endobugula sertula”, the uncultivated microbial symbiont of the marine bryozoan bugula neritina. J. Nat. Prod..

[7] Abe T., Kukita A., Akiyama K., Naito T., Uemura D. (2012). Isolation and structure of a novel biindole pigment substituted with an ethyl group from a metagenomic library derived from the marine sponge halichondria okadai. Chem. Lett..

[8] Yang X.-L., Wakimoto T., Takeshige Y., He R., Egami Y., Awakawa T., Abe I. (2013). Indole-porphyrin hybrids produced by metagenomics. Bioorg. Med. Chem. Lett..

[9] Fujita M.J., Kimura N., Sakai A., Ichikawa Y., Hanyu T., Otsuka M. (2011). Cloning and heterologous expression of the vibrioferrin biosynthetic gene cluster from a marine metagenomic library. Biosci. Biotechnol. Biochem..

[10] Kameyama T. (1987). Bisucaberin, a new siderophore, sensitizing tumor cells to macrophage-mediated cytolysis. I. Taxonomy of the producing organism, isolation and biological properties. J. Antibiot..

[11] Fujita M.J., Kimura N., Yokose H., Otsuka M. (2012). Heterologous production of bisucaberin using a biosynthetic gene cluster cloned from a deep sea metagenome. Mol. Biosyst..

[12] Fujita M.J., Sakai R. (2013). Heterologous production of desferrioxamines with a fusion biosynthetic gene cluster. Biosci. Biotechnol. Biochem..

[13] Ledyard K.M., Butler A. (1997). Structure of putrebactin, a new dihydroxamate siderophore produced by shewanella putrefaciens. JBIC J. Biol. Inorg. Chem..

[14] Fujita M.J., Nakano K., Sakai R. (2013). Bisucaberin b, a linear hydroxamate class siderophore from the marine bacterium tenacibaculum mesophilum. Molecules.

[15] Boettcher T., Clardy J. (2014). A chimeric siderophore halts swarming vibrio. Angew. Chem. Int. Ed..

[16] Schwyn B., Neilands J.B. (1987). Universal chemical assay for the detection and determination of siderophores. Anal. Biochem..

[17] Kadi N., Oves-Costales D., Barona-Gomez F., Challis G.L. (2007). A new family of atp-dependent oligomerization-macrocyclization biocatalysts. Nat. Chem. Biol..

[18] Kadi N., Song L., Challis G.L. (2008). Bisucaberin biosynthesis: An adenylating domain of the bibc multi-enzyme catalyzes cyclodimerization of n-hydroxy-n-succinylcadaverine. Chem. Commun. (Cambridge, UK).

[19] Kadi N., Arbache S., Song L., Oves-Costales D., Challis G.L. (2008). Identification of a gene cluster that directs putrebactin biosynthesis in shewanella species: Pubc catalyzes cyclodimerization of n-hydroxy-n-succinylputrescine. J. Am. Chem. Soc..

[20] Kim C.-M., Park Y.-J., Shin S.-H. (2007). A widespread deferoxamine-mediated iron-uptake system in vibrio vulnificus. J. Infect. Dis..

