# Supplementary material for: Production of Avaroferrin and Putrebactin by Heterologous Expression of a Deep-Sea Metagenomic DNA
Source: Mar Drugs. 2014 Sep 12;12(9):4799–809. doi: 10.3390/md12094799 (PMC4178481; doi:10.3390/md12094799)
Supplement: Supplementary File 1 [file marinedrugs-12-04799-s001.pdf]

Supplementary Information

Figure S1. Results of CAS assay.
Figure S2. Selected ion current LC-MS chromatogram of the culture broths.
Figure S3. ^{1}H NMR spectrum of avaroferrin (**1**) in CD_3OD + DMSO-d_6.
Figure S4. ^{13}C NMR spectrum of avaroferrin (**1**) in CD_3OD + DMSO-d_6.
Figure S5. High Resolution ESI MS spectra of avaroferrin (**1**).
Figure S6. ^{1}H NMR spectrum of putrebactin (**3**) in CD_3OD + DMSO-d_6.
Figure S7. High resolution ESI MS spectra of putrebactin (**3**).
Table S1. Amino acid sequence homology and most abundant products.
S1. DNA sequence of synthetic *bibC*^{C} gene.
S2. DNA sequence of synthetic *pubC* gene.

Figure S1. Chrome azurol S assay results of compound **1–3**. All experiments were performed triplicate and values are average of three (error bar: SD).

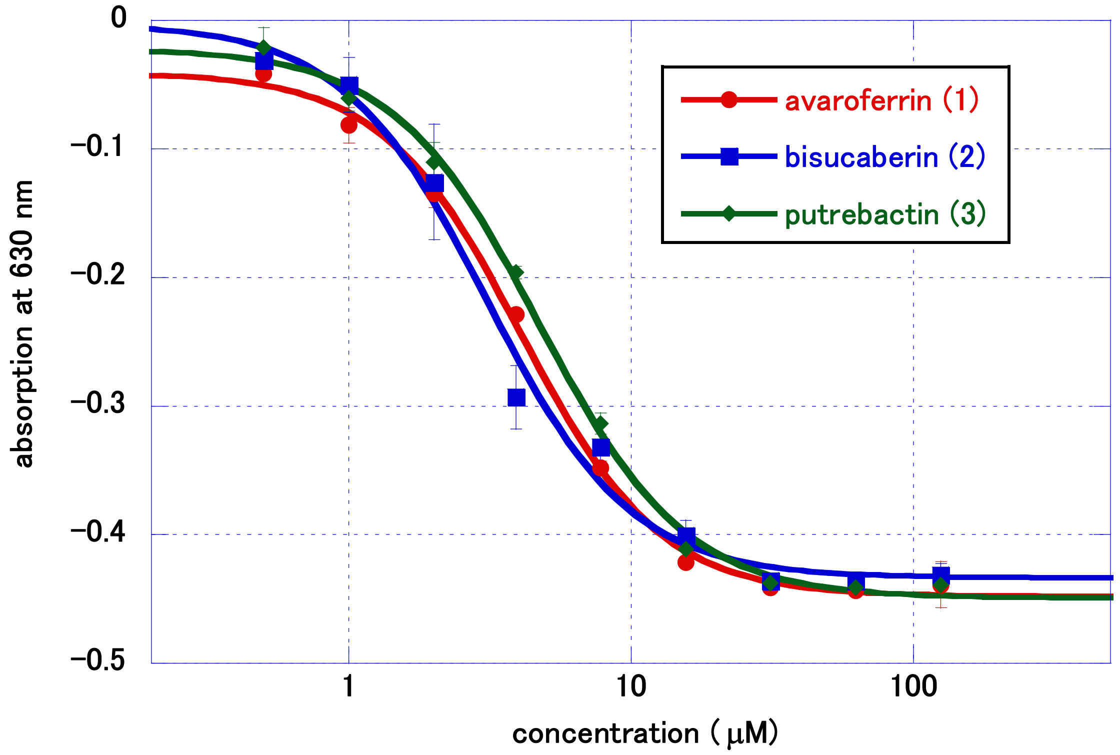

Figure S2. Selected ion current LC-MS chromatogram of the culture broths of the marine metagenome originated *mbsA-C* clone (**a**) and vector only clone (**b**). *MbsA-C* clone exhibited ion peaks of compounds **4** and **5**, while peaks corresponding to condensed products **1–3**, and **6–8** were absent.

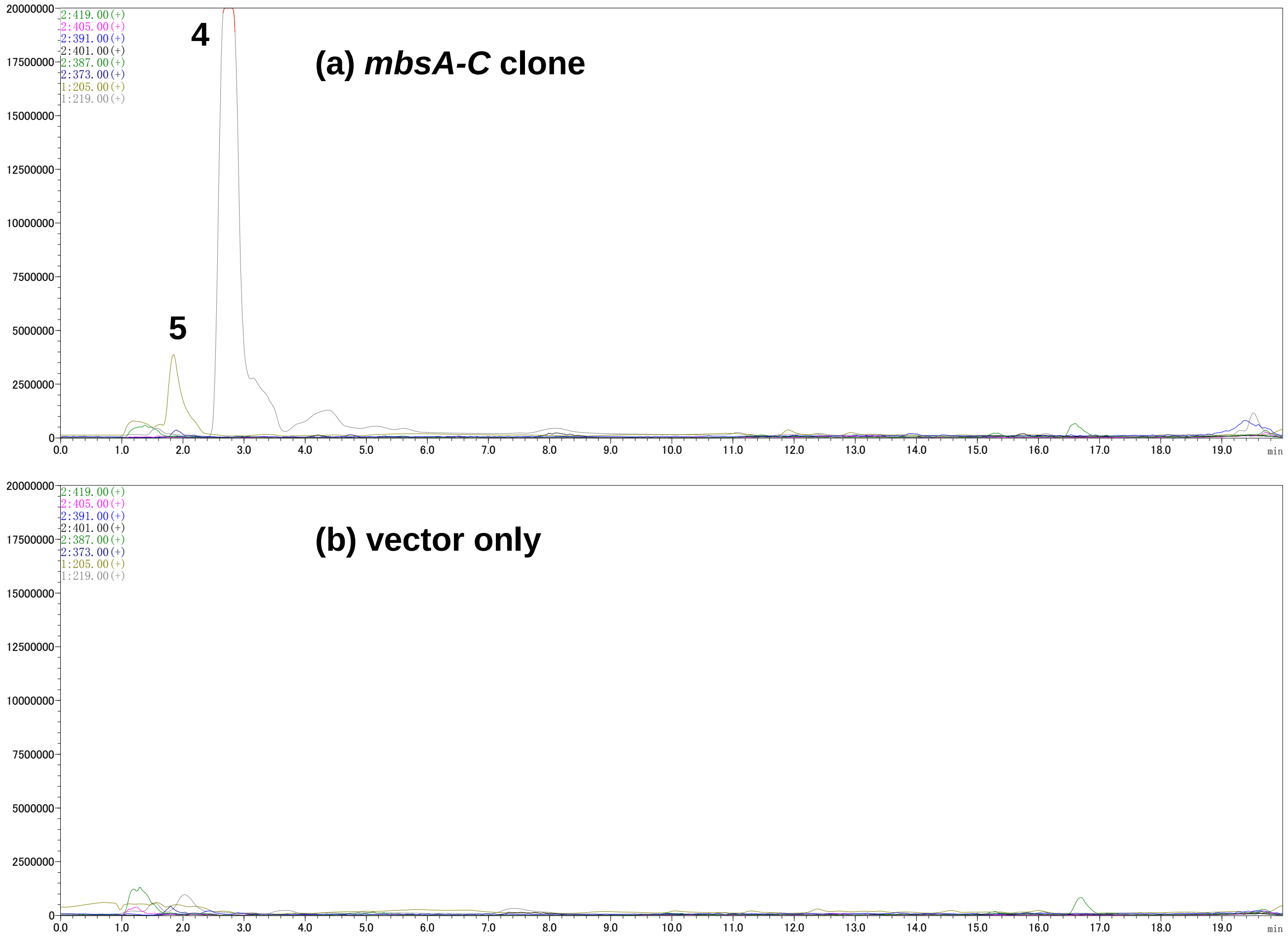

Figure S3. ^{1}H NMR spectru of avaroferrin (**1**) in CD_3OD + DMSO-d_6.

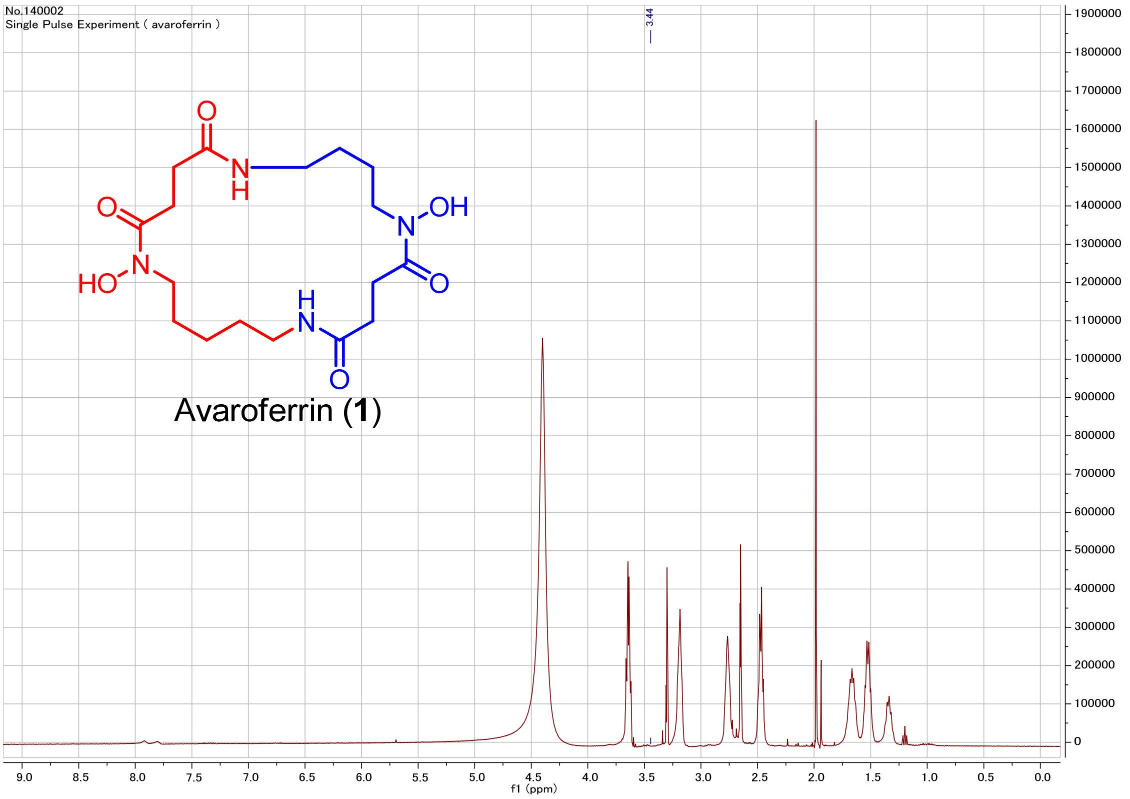

Figure S4. ^{13}C NMR spectrum of avaroferrin (**1**) in CD_3OD + DMSO-d_6.

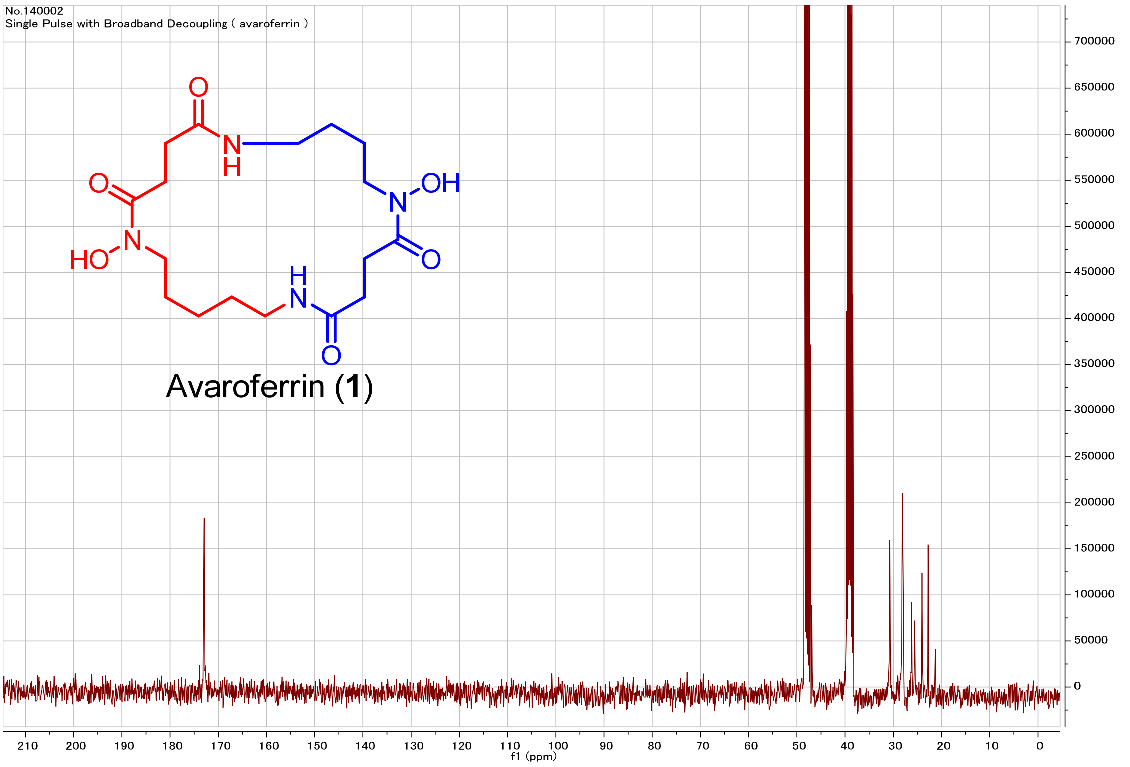

Figure S5. High Resolution ESI MS spectra of avaroferrin (**1**).

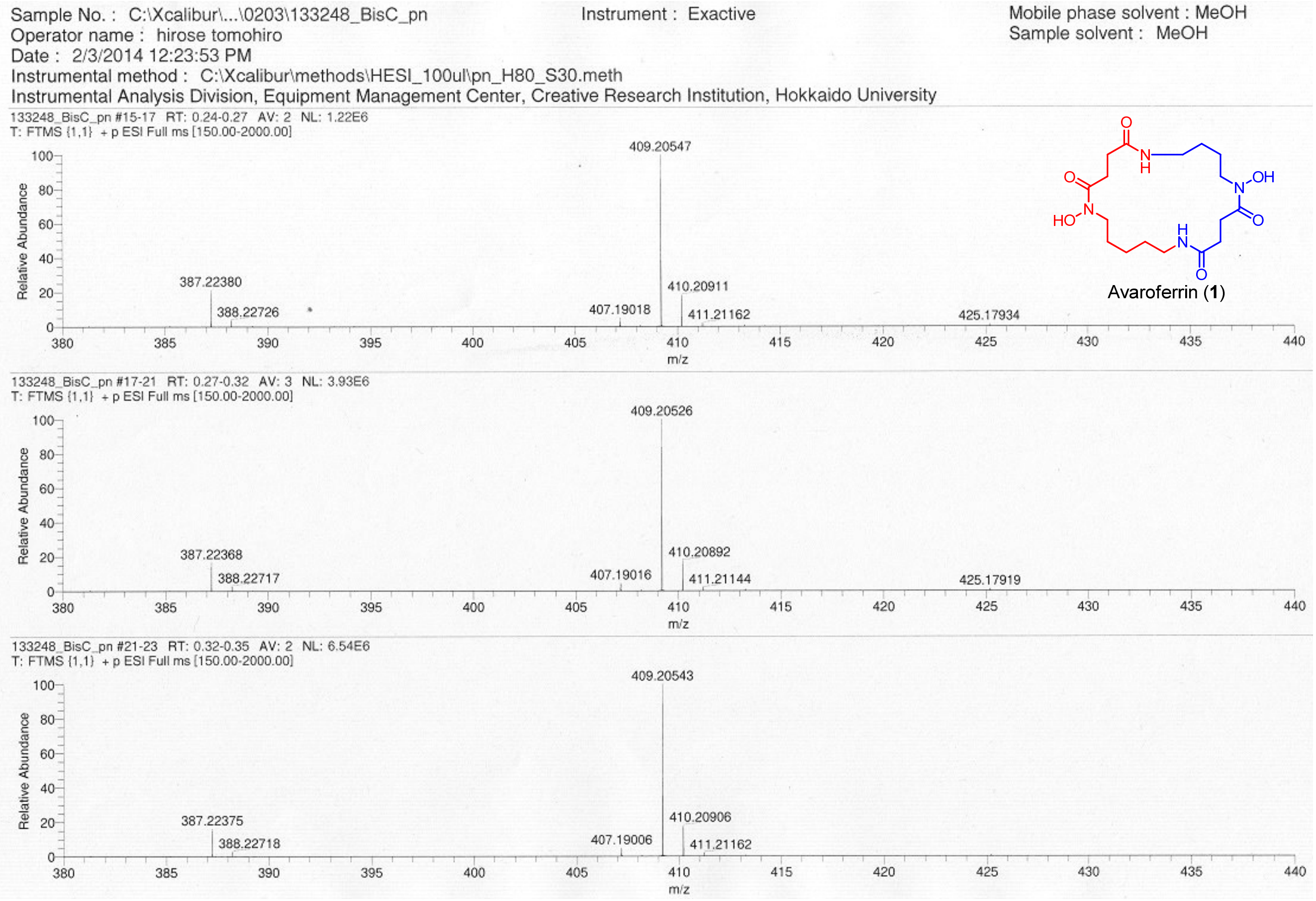

Figure S6. ^{1}H NMR spectrum of putrebactin (**3**) in CD_3OD + DMSO-d_6.

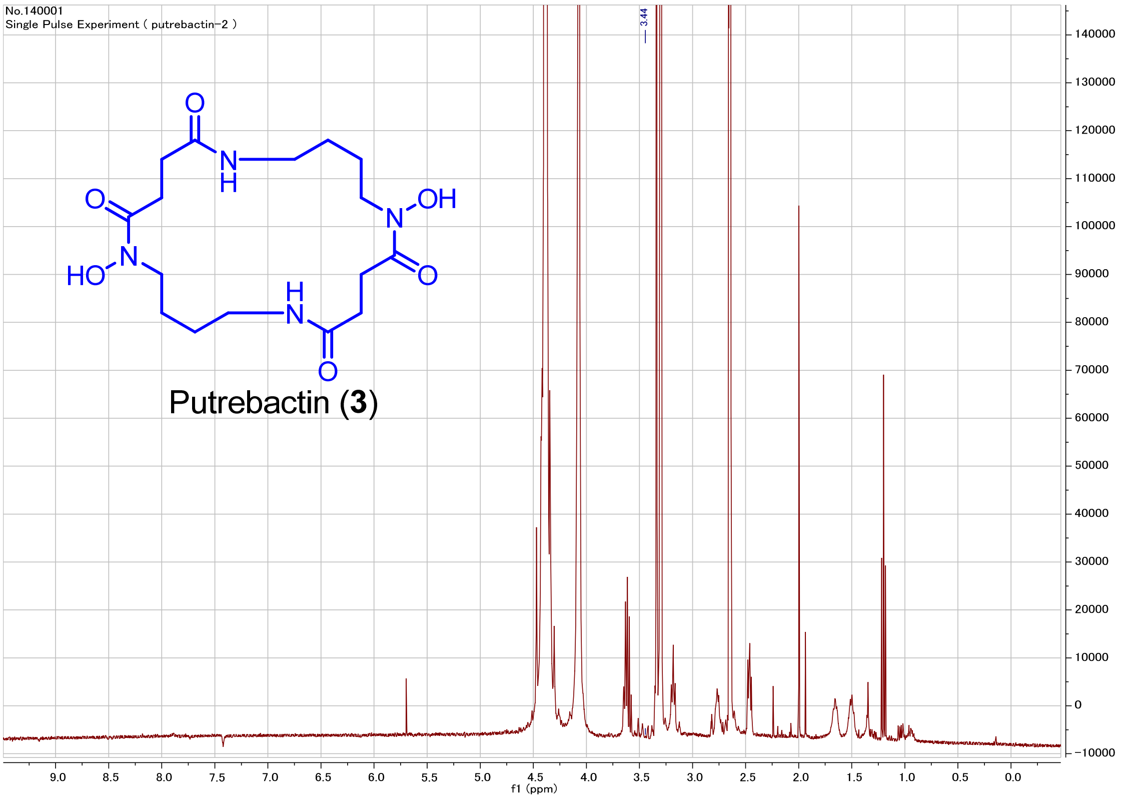

Figure S7. High resolution ESI MS spectra of putrebactin (**3**).

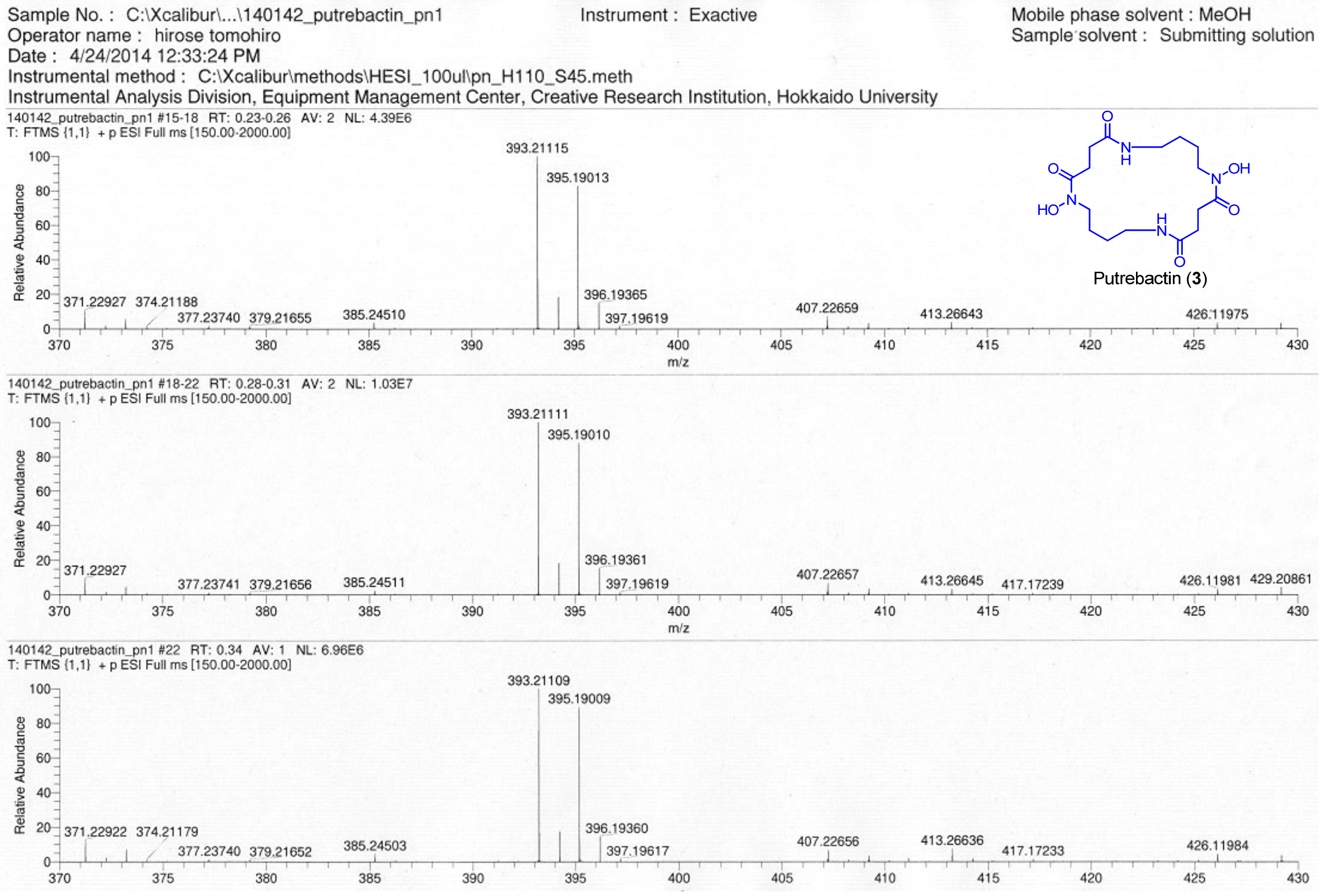

Table S1. Amino acid sequence homology and most abundant products.

	**BibC^C**	**PutC**	**Major Product**
MbsD	63%	64%	bisucaberin (**2**)
BibC^C		59%	bisucaberin B (**7**)
PutC			putrebactin (**3**)
AvaD^a			avaroferrin (**1**)

AvaD^a: sequence data not shown.

S1. DNA sequence of synthetic *bibC^C* gene

GTCGACATGAAGAATAGCAGCAAGAATCCAAGTTTGAGCTTGGCCACGTCACATCTGACC
ACTGAATACTGGCACAAGGCAAATCAACATCTGATCGCAAAGATGATCACGGAACTGTCGC
ACGAACAGATCATAACACCGATAAAGTTAGACGACGCGTCAAACGCCCAAGCTGCTAGTT
GGTGTATAACCTTTAACTCTGATACCGGTACCTCAGAGTATTTGTTCCGCGCTCGGCAATAC
CAACTGGATCATCTGTTCGTAGAGCCTCAATCCATTACCTGTACTAAGGATGATAAGAACCA
ACCGCTGGATGCCGTTTCCTTTATCCTGAGTTGTAGACATTTGCTTGAAATTAGTGACGCGT
TGCTTCCGACGTACCTGGAAGAGATTACGAGTACGTTATATAGCAAAGCCTATAAGCTTATG
CATCAAAATAAGACCTCCGCCCAATTAGCCAACGCGTCATACCAAGAGATAGAAGCAGCAA
TGACAGAAGGACATCCCGTTTTCATAGCGAATAATGGAAGAATAGGTTTCGATATGTTAGAC

CACGTGGAATTTAGCCCTGAATCAGGTCAGTCCCTGAACTTACAGTGGATCGCAGTGTTAA
GAGAGAAGACCTCCTTTGCGGTTATAGAATCACTGTCGTACGATAGACTTATCTTCGATGAA
TTGGGTCAGTCTCAGCTTAACGAATTCAACCAGCAACTGTCGATGCAGGGTTTAGAACCCT
CTCACTACTACCTTATGCCTATCCACCCGTGGCAATGGAGAGAAAAGATTTCACGGATATTC
GCGGCGGATATTGCAAACCAGTATGTAGTGCCATTGGGAACTACGGAAGATAAGTATCAAG
CACAGCAGAGTATTCGGACTTTCTTCAATTTAAGCTCGCCGGAGAAATGTTACGTGAAGAC
GGCGCTTTCTATTCTGAATATGGGTTTCATGCGTGGGCTTTCTCCTTATTATATGAGCCGGAC
CCCGGCTATTAACACCTTCATCGCAAATTTGATTGAGACTGACCCGTACTTCGCTAAGAAGC
AGTTCTTCGTTTTAAAGGAAGTAGCAGCGATCGGCTACCACCATTCCTACTACGAGCAAGC
TACGCGGACTGATAATCCGTATAAGAAGATGCTGTCTAGTCTTTGGCGGGAGTCGCCCTATG
CTCCAGATCAACACGGAAATGTACTGGTGAACAAGCAGCAGAAATTGTTAACTATGGCCTC
CTTGTTGCATGTGGATGATCAGGGAAAATCACTGATATCAGCCCTGATGGCCGACTCTCCAC
TTAGTGATCACAACTGGCTGAAGCAGTACATGGATTTATACTTGCAACCACTGCTGCATAGC
TTCTTTGCCTATGATTTGGTTTTCATGCCCCATGGTGAGAATCTGATTCTGGTATTAGAGGAC
AATTCTCCCATCAAGATAATTATGAAGGATATCGGAGAAGAGGTGGCGATCCTTAACGGGG
AAAAGACACTTCCAAACGATATGAACTGCTTGGCAGTCGATCTGGAAGACCCTATGAAACT
GAACTACATCTTGCTGGATATATTCGATTGTATCTTCCGCTTCATAGCACCTTTACTGGAGCA
ACAGACTCAAGTTTCAGAGAGCGACTTCTGGGAAATCGTTGCTGATTCAGTCAAGGATTAC
CAACAGGAGCATCCGCAGTTTGATGCGAAATATCAGCGGTACGATTTGTACTGCTCTAGCTT
CGCGCGGACATGTTTGAATCGTATCCAGCTTAATAATAATCAGCAAATGATCGACCTGGAGG
ATAGAGAGAAGAATCTTAGATTTGCCGAGGATATCGCAAATCCACTGGCATTGTTTGCTAAG
ACACATCGTATCATCGGGCCC

S2. DNA sequence of synthetic *pubC* gene

GTCGACATGAATCTGGCAACCAATCGTGCGTTACTGAAGCCGTTTACGCAGGCCGAGTTTC
CGACTCTGGAAGCGGCAGCACATCCGCATGTTCCGGCACATTTGATGCCGGAATACTGGCA
AGCAGCAAATCGCCATCTGGTGAAGAAAATCCTGTGTGAATTCACCCACGAGAAAATTATC
AGCCCGCAAATCTATCGTCAAGCAGCCGGGATTAACCACTATGAACTGCGCCTGAAAGATT
GCACCTATTACTTCTCGGCCCGCCATTATCAATTGGATCATCTCGAAATTGAGGCTGGTAGC
ATCCGCGTGTCAAGTGCGGGCCAGGATAAACCGCTGGATGCGATGTCCTTAATTATCAAGC
TGAAAGACGCTCTGGGTATGTCAGAGACATTACTGCCGACCTATCTTGAGGAAATCACCTC
GACCCTTTACAGCAAAGCTTACAAATTGGCGCATCAAGCGATTCCTGCTACAACTCTGGCG
AAAGCGGATTATCAGACGATTGAGGCGGGTATGACGGAAGGACACCCAGTTTTCATTGCGA
ACAATGGTCGTATTGGCTTTGACATGCAGGATTATGATCAGTTTGCACCAGAAAGCGCTTCG
GCACTCCAGTTGGTTTGGATTGCGGTTCGCAAAGACAAGACCACCTTCAGCTCTTTGGAA
GGTCTGGATCATGACTCTCTGCTTAAACAGGAACTGGGTGAACAGTTCACGAAATTTCAGC
AGCACCTGTCGGCTTTAGGTCAAGCGGCAGATTCGTTTTACTTCATGCCGGTTCACCCATG
GCAGTGGCGTGAGAAAATTGCCCGTACATTTGCCGGCGAAATTGCGCGTGGGGATATCATT
TATCTGGGCGAAAGTCAGGACTGTTACCAGGTCCAACAGTCGATCCGGACCTTCTTCAACT
TATCTGCGCCACAGAAGTGCTACGTGAAAACCGCCCTGAGCATCCTCAACATGGGGTTTAT
GCGTGGCTTATCTCCGCTGTATATGAGCTGTACGCCCCAGATCAACGCTTGGGTTGCGGATC

TCATTGAATCCGACTCCTATTTTGCGGAACAAGGCTTCGTCATTCTCAAAGAAATCGCAGCC
ATTGGTTATCACCATCGCTACTATGAGGAAGCATTGACTCAAGACTCAGCTTACAAGAAAAT
GCTTTCTGCGCTGTGGCGCGAGTCCCCTCTGCCGCATATTGAACCACAGCAAACGCTGATG
ACGATGGCTGCCTTACTGCATGTGGACCACCAAGAACAGGCCTTGTTGGCCGCGCTGATCA
AACACAGTGGCTTAAGCGCGAAAGAATGGGTCAAACGGTATCTGAATCTGTATCTGAGTCC
GTTGCTGCACGCTTTCTTTGCCTACGATCTTGTCTTTATGCCTCATGGCGAAAACCTTATTCT
GGTCTTGGATGCAGGCATTCCCGTGAAAATCCTGATGAAAGACATTGGAGAGGAAGTAGC
CGTGCTGAACGGGTCCGAACCCCTCCCGCAAGAGGTACAGCGTCTGGCGGTGGAACTTGA
AGAAGAGATGAAACTGAACTACATTCTCCTGGATATCTTTGATTGCATTTTCCGCTATCTTGC
CCCGATTCTGGATAAACAGACCGAAGTGAGTGAGGCGCAGTTTTGGGAATTAGTAGCGGAT
AATGTGCGTGATTACCAAGCCCAGCATCCGCAGTTAGCCGACAAGTTCGCTCAATATGACC
TGTTCAAAGATAGCTTTGTACGCACTTGCCTGAATCGCATCCAGCTCAACAACAACCAGCA
AATGATCGATCTGGCCGATCGCGAAAAGAATCTGCGCTTTGCGGGTGGCATTGACAATCCT
CTGGCCGCATTTCGCCAGTCACACGCCTTTGGAAACCAGAAACTGAAACCCAAAAGCGGG
CCC
